# An advanced shape-fitting algorithm applied to quadrupedal mammals: improving volumetric mass estimates

**DOI:** 10.1098/rsos.150302

**Published:** 2015-08-19

**Authors:** Charlotte A. Brassey, James D. Gardiner

**Affiliations:** 1 Faculty of Life Sciences, University of Manchester, Manchester M13 9PL, UK; 2 School of Computing, Science and Engineering, University of Salford, Salford M5 4WT, UK

**Keywords:** α-shapes, body mass, volumetric, fossil, *Megatherium*, *Mammuthus*

## Abstract

Body mass is a fundamental physical property of an individual and has enormous bearing upon ecology and physiology. Generating reliable estimates for body mass is therefore a necessary step in many palaeontological studies. Whilst early reconstructions of mass in extinct species relied upon isolated skeletal elements, volumetric techniques are increasingly applied to fossils when skeletal completeness allows. We apply a new ‘alpha shapes’ (*α*-shapes) algorithm to volumetric mass estimation in quadrupedal mammals. *α*-shapes are defined by: (i) the underlying skeletal structure to which they are fitted; and (ii) the value *α*, determining the refinement of fit. For a given skeleton, a range of *α*-shapes may be fitted around the individual, spanning from very coarse to very fine. We fit *α*-shapes to three-dimensional models of extant mammals and calculate volumes, which are regressed against mass to generate predictive equations. Our optimal model is characterized by a high correlation coefficient and mean square error (*r*^2^=0.975, m.s.e.=0.025). When applied to the woolly mammoth (*Mammuthus primigenius*) and giant ground sloth (*Megatherium americanum*), we reconstruct masses of 3635 and 3706 kg, respectively. We consider *α*-shapes an improvement upon previous techniques as resulting volumes are less sensitive to uncertainties in skeletal reconstructions, and do not require manual separation of body segments from skeletons.

## 1. Introduction

To paraphrase Theodosius Dobzhansky: ‘Nothing in biology makes sense except in the light of [body mass]’ [1, p. 125]. Body mass is arguably the most fundamental property of an organism, and key evolutionary concepts within the fields of ecology, physiology and biomechanics can only be understood within its context. The interpretation of important evolutionary transitions such as the origin of endothermy [2] and avian flight [3], and ecological ‘rules’ such as Cope's rule (an increase in body size over evolutionary time [4]) and Bergmann's rule (an increase in body size with decreasing temperature [5]), rely heavily upon knowledge of body mass. Consequently, calculating the body mass of extant and extinct species is of broad interest across biological disciplines.

When estimating body mass, palaeobiologists typically proceed by measuring properties such as tooth and bone dimensions from an extant interspecific sample, and calculating their relationships to body size ([6], and references therein). The equations derived can subsequently be used in a predictive capacity: commonly applied metrics include craniodental measures applied to fossil ungulates [7], femoral head breadth in fossil hominids [8], and humeral and femoral circumference applied to non-avian dinosaurs [9]. The ability to predict fossil body mass on the basis of isolated skeletal elements is potentially very useful, but also warrants caution. The fossil record is extremely fragmentary, and the vast majority of species throughout Earth's history are known only from partial skeletons. However, by basing mass estimates on single linear dimensions, important palaeoecological studies of broad taxonomic and chronologic scope can be undertaken, that would otherwise be unachievable if fragmentary remains were excluded [10].

Concerns, however, have been raised regarding the application of traditional bivariate linear equations to predict fossil body mass. If a fossil species possessed unusually proportioned skeletal features, relative to the extant dataset from which the predictive equation was derived, then these robust or gracile features bias any resulting mass estimates. As an exaggerated hypothetical example, the elongate canines of the sabre-toothed cat (*Smilodon fatalis*) would hint at a much larger animal than is realistic if considered in isolation from the rest of the preserved skeleton. Furthermore, when basing mass estimates on fragmentary remains, there may be some difficulty in assigning an ontogenetic status to the individual, thus increasing the risk of confounding interspecific scaling with ontogenetic scaling [11].

For these reasons, volumetric techniques have become an increasingly popular method to estimate body mass of extinct species, when completeness of the fossil skeleton allows. Early volumetric models involved physically sculpting clay scale models [12–14] and estimating volume via fluid displacement. However, alongside advances in computer graphics, there has been a shift towards digital shape-fitting as a means of estimating skeletal volume, and subsequently body mass. Initial mathematical techniques [15–17] worked on two-dimensional traced outlines of animals, from which mass may be calculated either by subdividing the body into a series of simple transverse slices, or by describing the basic body shape using a series of polynomial equations. By taking such an approach, important properties such as centre of mass (COM) and segment inertial parameters (necessary for the dynamic analyses of motion) can be estimated in addition to total body mass. Qualitative reconstructions of the soft tissue outline are required however and are open to the subjective interpretation of the researcher.

Alternatively, shapes can be fitted around the skeleton within computer-aided design software packages to approximate the soft-tissue contours of an animal. Previous approaches have ranged from fitting simple geometric primitives, such as spheres and cylinders [18] and other rotational solids [19], to more complex non-uniform rational B-splines (NURBS) [20,21]. NURBS are polynomial functions used to define smooth irregular curves and surfaces that may be fitted around data points. B-spline solids can be easily manipulated via ‘control points’, allowing for the reconstruction of a soft-tissue body outline from skeletal parts alone and for the embedding of low-density objects (e.g. lungs, trachea) inside the body cavity [22]. NURBS-based modelling techniques may be thought of as the digital equivalent of clay sculpting, with the added advantages of improved repeatability and shareability. The initial application of NURBS to an extinct species involved the shrink-wrapping of polygonal hulls around landmark points digitized from the skeleton of a *Tyrannosaurus rex* [20]. Models were subsequently manipulated using via points to replicate the ‘fleshed-out’ appearance of the animal. The extent of the reconstructed soft-tissue contour, however, is open to interpretation and time-consuming sensitivity analyses are often required to quantify the effect of uncertainties in soft-tissue volume, lung volume and body density on total body volume, and hence body mass [20,21,23–25].

**Figure 1.**
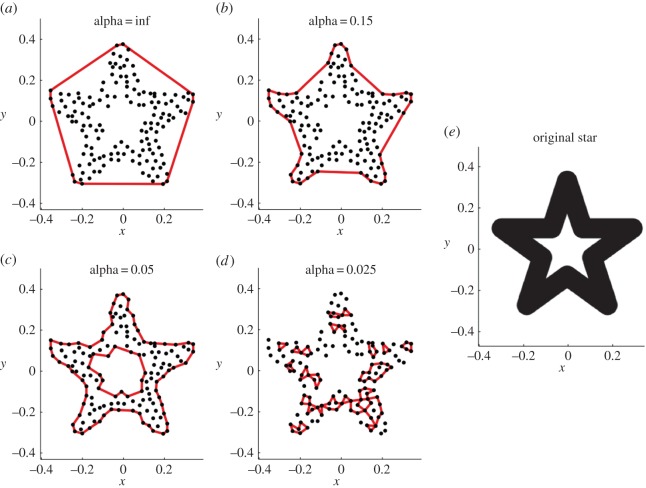
A ‘family’ of *α*-shapes for a given two-dimensional dataset. (*a*) The special case ‘convex hull’ *α*-shape calculated when *α* is infinite; (*b*−*d*) *α*-shapes calculated as the radius *α* decreases relative to the point spacing of the dataset; (*e*) original ‘solid’ geometric shape upon which point cloud (*a*−*d*) was derived. The suite of *α*-shapes ranges from ‘crude’ to ‘fine’ representations of the two-dimensional dataset.

Recently, a mass-estimation technique has been put forward which combines aspects of three-dimensional volumetric modelling and traditional linear bivariate predictive equations, by using ‘convex hulls’. A convex hull can intuitively be thought of as a shrink-wrap polytope around a given set of points, such that no points exist outside of the shape (figure 1*a*). Sellers *et al.* [26] apply convex hull fits to segmented three-dimensional models of modern quadrupedal mammal skeletons in order to determine a value for minimum convex hull volume (*C*_vol_) of the animal as defined by the bony limits of its skeleton. *C*_vol_ can subsequently be used as the independent variable in a predictive equation with body mass. It must be emphasized that convex hulling as applied here and by Sellers *et al.* [26] to articulated skeletons *does not* seek to recreate the original fleshed-out form of the animal and should not be confused with other digital sculpting techniques. Rather *C*_vol_ is simply used an alternative metric of skeleton size (analogous to using femur circumference or molar height) to be incorporated into a bivariate predictive equation and is of uncertain use in the calculation of COM and segment inertial properties from skeletons (although see [27] for application of convex hulls to fleshed out anthropometric datasets).

Compared to traditional bivariate predictive equations based on single limb or cranial dimensions, convex hulling incorporates a far greater amount of information from the skeleton into a mass estimate and is therefore less liable to biasing by isolated robust or gracile bony elements. For instance, Brassey *et al.* [28] apply a ratite-based convex hull equation to extinct dinornithiform birds (a group known to possess distinctive robust limb bones) and revise down previous body mass estimates. In addition, unlike previous digital sculpting techniques, the fitting of convex hulls around an articulated skeleton requires no subjective decisions regarding the volume of soft tissues to be reconstructed. For example, Sellers *et al.* [29] estimate the mass of one the largest known terrestrial vertebrates, the sauropod dinosaur *Argentinosaurus huinculensis*, using a convex hull-fitting algorithm without the need for extensive sensitivity analyses.

The application of convex hulling to estimate the volume defined by a three-dimensional set of points does, however, incur some disadvantages. If the three-dimensional model possesses substantial concave areas, holes or tunnels, the convex hull is stretched across these regions, possibly resulting in the overestimation of volume (figure 1*a*). To circumvent this problem, Sellers *et al.* [26] subdivide the skeleton into anatomical segments (trunk, thigh, shank, etc.) in order to avoid the large area between the legs, for example, being included in the volume estimate. If the articulated skeleton possesses a flexed neck and/or tail, this requires further subdivision of the anatomical units at arbitrary locations in order to ensure ‘straight segments’ (see Sellers *et al*'s [26] electronic supplementary material, figure S4). Furthermore, the final extent of the convex hull is determined solely by the outermost points, with the vast majority of points lying inside the hull and not contributing to hull geometry. Therefore, any noise or error in the outermost points has an unduly large effect on the final convex hull volume and hence mass.

Here, we explore a shape-fitting technique known as ‘alpha shapes’ or ‘*α*-shapes’ as a potential improvement upon convex hulling. *α*-shapes are a generalization of the concept of convex hulls and are defined not only by the set of points to which they are fitted but also depend upon the value alpha (*α*) which can range between 0 and infinity [30]. By altering the value of *α* for a given set of points (*S*), a family of *α*-shapes is defined which can be thought of as ranging in refinement from ‘fine’ to ‘crude’, ending with a convex hull at sufficiently high values of *α* (figure 1*a*−*d*). To explain conceptually, consider a burrowing sphere of radius *α* passing through a three-dimensional volume comprising a series of solid rocks supported within a removable soil matrix. Wherever the distance between rocks is greater than the size of the sphere, the sphere can pass through and the soil is removed. The remaining unexcavated volume (‘soil’) is the *α*-shape of the given set of points (‘rocks’). It should be noted that regions of ‘excavated’ volume may lay completely inside the points and not connected to the outside (figure 1*c*). Once all the points defining the *α*-shape have been identified, they are connected by straight lines (rather than curves) (figure 1) and the area/volume calculated.

We use the term ‘*α*-shape’ to refer to all calculated alpha shapes *excluding* the special case convex hull calculated when *α* is infinite. Herein, we present a new volumetric technique, based on *α*-shapes, applicable to the problem of fossil body mass estimation, and compare our approach to the pre-existing convex hull methodology. We hypothesize that *α*-shape volumes of the vertebrate skeleton might be more reliably applied to the problem of mass estimation than convex hulls since:

(i) depending on the value of *α*, it is not necessary to subdivide the skeleton *a priori* into anatomical units in order to achieve a tight-fitting hull. Thus, any ambiguity associated with skeletal segmentation is avoided;
(ii) relative to convex hulls, a far greater number of points contribute to define the overall form of the *α*-shape, thereby minimizing the effect of a small number of potentially erroneous outlying points on total calculated volume; and
(iii) the parameter *α* can be modified, thus defining a suite of *α*-shapes for each skeleton. For a given interspecific dataset, a range of *α* values can therefore be explored and an optimal *α* identified upon which to derive a predictive model.

## 2. Material and methods

### 2.1 Model dataset

Three-dimensional point cloud models of 14 extant quadrupedal mammal skeletons first published by Sellers *et al.* [26] were sourced from http://www.animalsimulation.org. Sellers *et al.* [26] scanned the skeletons using a Z+F Imager -5006i LiDAR scanner at the Oxford University Museum of Natural History (OUMNH). Values for body mass were not available for the mounted skeletons and were therefore assigned using literature-derived scaling equations (table 1) [26]. In order to test our predictive models on extinct taxa, a three-dimensional model of the composite skeleton of a woolly mammoth *Mammuthus primigenius* (USNM 23792), scanned using a LiDAR system was sourced from the Smithsonian Museum's ‘X 3D’ website (http://3d.si.edu). In addition, a point cloud model of an articulated composite cast of a giant ground sloth *Megatherium americanum* (NHMUK 26540), on public display in the Natural History Museum (NHM), London, was generated using the photogrammetric technique outlined elsewhere [31,32] and the software ‘Photoscan’ (Agisoft, St Petersburg, FL, USA). The *Megatherium* three-dimensional point cloud model is available by request from the curator of fossil mammals (NHM). In the case of the OUMNH dataset, the antlers of some cervid specimens were missing from the skeleton and we therefore excluded all antlers and tusks from the analysis of OUMNH and USNM specimens. The *α*-shape technique presented here is, however, highly applicable to species possessing unusual and irregularly shaped accessory structures such as these (see Discussion).

**Table 1.** Extant dataset of articulated skeletons sourced from the Oxford Museum of Natural History, and their calculated volumes using convex hull and alpha shape-fitting techniques. (Values for *C*_vol_ and *α*_vol_ are calculated from skeletons comprising 500 000 points each. Values for *C*_vol(sub)_ are taken directly from Sellers *et al.* [26].)

species	mass (kg)	*C*_vol_ (m^3^)	*C*_vol(sub)_ (m^3^)	*α*_vol_ (m^3^)
*Bison bison*	558.5	1.573	0.473	0.555
*Bos taurus*	323.7	0.666	0.219	0.277
*Camelus dromedaries*	427.0	1.436	0.332	0.434
*Cervus elephus*	89.5	0.435	0.084	0.114
*Dicerorhinus sumatrensis*	470.3	1.062	0.363	0.466
*Elephas maximus*	2352.0	5.107	2.093	2.287
*Equus caballus*	517.5	1.525	0.370	0.467
*Giraffa camelopardalis*	638.2	2.479	0.447	0.612
*Loxodonta africana*	2734.9	7.005	2.748	3.330
*Megaloceros giganteus*	435.6	1.199	0.301	0.361
*Rangifer tarandus*	95.8	0.394	0.076	0.123
*Sus scrofa*	107.4	0.257	0.079	0.108
*Tapirus indicus*	295.3	0.530	0.172	0.230
*Ursus maritimus*	206.1	0.367	0.111	0.159

### 2.2 Shape-fitting techniques

The OUMNH dataset was imported into MATLAB as a series of ASCII files, each comprising a point cloud skeleton of one individual saved as *x*-, *y*-, *z*-coordinates. Unlike Sellers *et al.* [26], we do not subdivide the skeletons into anatomical units and instead fit shapes to the entire skeleton. Convex hulls and *α*-shape were fitted using the ‘alphavol’ package available from MATLAB Central file exchange written by Jonas Lundgren (http://www.mathworks.co.uk/matlabcentral/fileexchange/28851-alpha-shapes), which calculates both the fit of the *α*-shape and its associated volume (*α*_vol_).

The final volume of a three-dimensional *α*-shape is a function of both the points to which it is fitted *and* the value of *α* defining the refinement of the fit (i.e. crude to fine). The mammals included in our extant dataset span a large range of body sizes, from the African elephant (*Loxodonta africana*) to the wild boar (*Sus scrofa*). If a global value for *α* were used to calculate the *α*-shape for both skeletons, it would result in a ‘finer’ fit for the elephant than the wild boar as the distance between skeletal features (e.g. rib spacing) scales with the size of the animal. It was therefore necessary to normalize the value of *α* to the overall size of each animal to ensure a consistent ‘refinement’ of fit across the animals. We calculated a relative alpha for each animal as:

$$\alpha =l_{\mathrm{ref}}\times k,$$

where *l*_ref_ is an animal-specific reference length calculated as the mean distance of each point in the skeleton from the centroid, and *k* is the ‘refinement coefficient’ which defines the fit of *α*-shape from ‘crude’ to ‘fine’. *α*_vol_ was calculated for each skeleton at 200 values of *k* ranging between 0.01 and 100. It is important to note that while the *α*_vol_ at certain values of refinement coefficients is likely to closely match the physiological volume of the animal, we are *not* attempting to directly calculate the refinement coefficient that most closely produces physiological volume, and from that calculate mass. Rather we are investigating *α*_vol_ at various degrees of refinement as a correlate for body mass (similar to previous models based on femoral length or cross-sectional area). Indeed, the *α*_vol_ which best correlates (i.e. highest *r*^2^) to body mass may be significantly larger or smaller than physiological volume.

There is no *a priori* reason why ‘mean distance to centroid’ ought to be the optimal method for size-normalizing *α*. We therefore also investigated other skeletal dimensions as potential metrics for mass-normalization, such as maximum height, maximum length and length of bounding box diagonal, all of which may be non-subjectively extracted from the models. In addition, we also investigated shoulder height and hip height as potential metrics, both of which require some user intervention to determine the location at which measurements are taken.

Furthermore, the OUMNH skeletons were derived from a single detailed LiDAR scan of a section of the main gallery of the museum. Individual skeletons were subsequently isolated out from the original large LiDAR scan, and as such the *absolute point density* was constant across the sample. It follows that the point clouds representing the skeletons of smaller mammal species (wild boar, *n*=535 819) comprise less points than those of larger species (African elephant, *n*=7 760 648), and therefore point density *relative to overall body size* decreases in smaller species. We therefore normalized the dataset such that each skeleton comprised 500 000 points (randomly downsampled to approximately equal the number of points comprising the smallest individual, *S. scrofa*). We also generated several additional normalized datasets of skeletons subject to downsampling (number of points per skeleton ranging from 250 000 to 1000) in order to investigate the potential effect of point cloud density upon the relationship between *α*_vol_ and body mass. Random downsampling, by definition, tends to produce variable results as each repeated run will not return exactly the same points for each skeleton. Therefore, to deal with the variable results that downsampling produces, the analysis was repeated 10 times and average values taken.

Ordinary least-squares (OLS) regressions of natural log-transformed *α*_vol_ against body mass were calculated for every iteration of *k* (i.e. from coarse to fine fits). The regression model with the highest *r*^2^ was selected as the ‘optimum’ predictive equation to calculate fossil body masses. The *α*_vol_ for both *Ma. primigenius* and *Me. americanum* were then calculated at optimum *k* and body masses predicted. It is again worth highlighting that the *k*-value at which *r*^2^ is highest may result in an *α*_vol_ that differs considerably from the physiological volume. The associated MATLAB file and data for the prediction of fossil body masses are available for download from Dryad (http://dx.doi.org/10.5061/dryad.ps80f). For those wishing to explore the *α*-shapes technique without using MATLAB, an *α*-shapes function is incorporated within the freely available software ‘Meshlab’ (http://meshlab.sourceforge.net). However, for the purpose of fitting numerous alpha shapes to multiple skeletons and subsequently conducting regression analyses, we consider a custom-written MATLAB script to be both quicker and less liable to user error than manual shape-fitting in Meshlab.

We chose not to apply phylogenetically corrected regression models here, as the original convex hull analysis of Sellers *et al.* [26] conducted on the same dataset found no appreciable difference in predictive models when accounting for phylogeny. Furthermore, the application of phylogenetically corrected equations to predicting the body mass of a species not included in the original model remains uncertain [33].

## 3. Results

**Figure 2.**
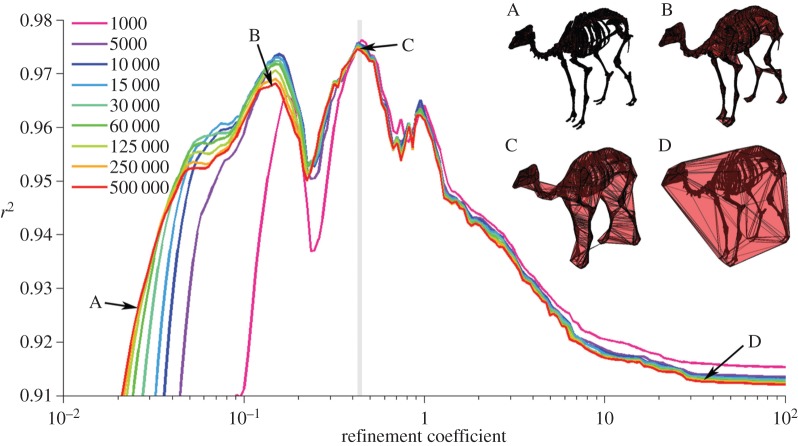
Variation in *r*^2^ against the refinement coefficient (*k*) for a series of downsampled skeleton datasets. *r*^2^ values derived from the natural-log transformed OLS regression of body mass (kg) against *α*_vol_ (m^3^). Grey shaded area represents narrow range in which optimal *k*-values (as defined by highest *r*^2^) occur for all datasets. Inset: four *α*-shapes fitted around the skeleton of *Camelus* comprising 500 000 points, illustrating increasing refinement coefficients from A to D. Arrows indicate position of each *α*-shape on the curve of *r*^2^ against *k*.

**Figure 3.**
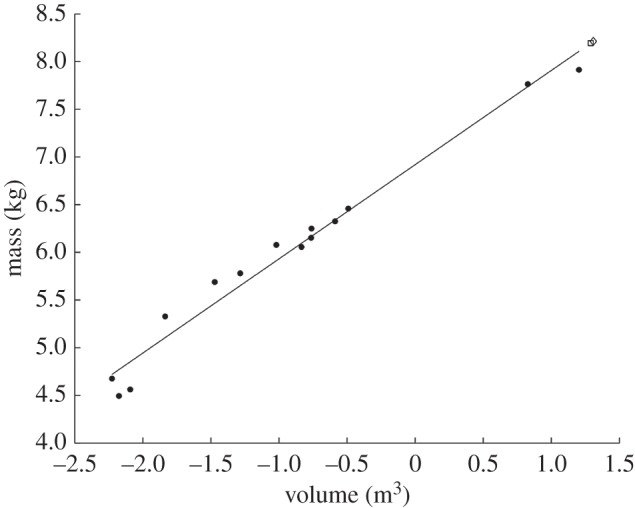
The relationship between *α*-shape volume and body mass for articulated skeletons comprising 500 000 points, for the ‘optimal’ *k*-value of 0.427. Extant taxa, filled circles; *Ma. primigenius*, open square; *Me. americanum*, open diamond.

When regressing log *α*_vol_ against log body mass for the densest point cloud dataset (animals represents by 500 000 points), the greatest *r*^2^-value of 0.975 occurs at a *k*-value of 0.427 (figure 2, red line). This refinement coefficient corresponds to an *α*-shape in which the left and right appendages within the forelimbs and within the hindlimbs are conjoined, but the forelimbs and hindlimbs remain distinct from one another (figure 2*c*). Additionally, the ribcage is entirely enclosed within the *α*-shape. The relationship between body mass and *α*-shape volume for the original dataset when *k*=0.427 is illustrated in figure 3. The constants of the equation describing this relationship can be found in table 2, as well as the results for the unsegmented convex hull equation and the segmented convex hull from Sellers *et al.* [26]. A second smaller peak occurs at a *k*-value of 0.1496 and an *r*^2^ of 0.9682, with a corresponding *α*-shape in which the fit passes within the ribcage and all limbs are distinct (figure 2*b*). When *k* exceeds 10, correlation coefficients plateau at values between 0.91 and 0.92 and the *α*-shapes tend towards a convex hull (figure 2*d*). Once *k*-values drop below 0.1, the *r*^2^-values of the regression tend to decrease rapidly as the alpha shapes start passing inside the bones and become a discrete set of disconnected volumes rather than one complete ‘shrink wrap’ of the skeleton. Calculated *α*-shape volumes (*α*_vol_) for the modern dataset (500 000 points per skeleton) are given in table 1 for the optimal *k*-value of 0.427, alongside convex hull volumes for the unsegmented skeletons (*C*_vol_) considered here and the segmented skeletons published elsewhere (*C*_vol(sub)_) [26].

**Table 2.** OLS of natural log body mass (kg) against natural log fitted shape volume (m^3^). (±95%, ±95% confidence intervals of the intercept and slope; m.s.e., mean square error of the regression.)

fit	*a*	*a*±95%	*b*	*b*±95%	*r*^2^	m.s.e.
*C*_vol_	5.906	5.723–6.096	0.995	0.800–1.190	0.912	0.087
*C*_vol(sub)_	7.103	6.981–7.211	0.926	0.854–0.993	0.979	0.021
*α*_vol_	6.920	6.781–7.059	0.988	0.888–1.088	0.975	0.025

**Figure 4.**
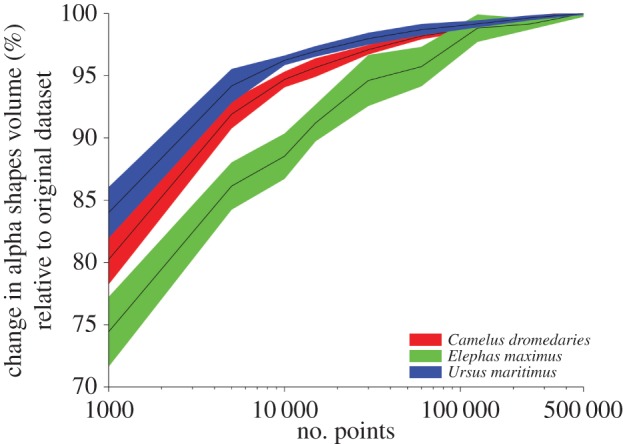
Change in volume relative to the original data (500 000 points per skeleton) with downsampling for three example skeletons. Coloured bands represent the standard deviation (10 repeats) either side of the averages (black lines).

Downsampled datasets (250 000 to 1000 points representing each skeleton) follow a remarkably similar trend to those observed for the densest point clouds (figure 2). Peak *r*^2^-values for all datasets fall within a narrow range of refinement coefficients (*k*=0.427−0.449, table 3), with the sparsest point cloud dataset (*n*=1000) producing the largest correlation coefficient of 0.976. The difference between peak *r*^2^-values derived from the densest (*n*=500 000) and maximum downsampled (*n*=1000) datasets is extremely small however (*r*^2^=0.975 and *r*^2^=0.976, respectively). We consider this minor effect to be owing to the variable impact of downsampling upon *α*_vol_. Calculated *α*-shape volumes decrease as point cloud density is reduced (figure 4), yet the *rate of change* is not constant across the sample. *α*_vol_ of *Elephas maximus* decreases much more rapidly than that of *Ursus maritimus* when subject to the same downsampling, for example, although this effect is not significantly correlated to body mass (*p*=0.09, *r*^2^=0.22) at *n*=1000. Additionally, standard deviation around mean *α*_vol_ increases as point cloud densities are reduced (figure 4).

**Table 3.** The effect of point density on optimal *k* (as defined by the highest calculated *r*^2^), the associated regression statistics and reconstructed volume and body mass of *Ma. primigenius* (USNM 23792) and *Me. americanum* (NHMUK 26540). (OLS regressions are natural log-transformed.)

no. points (1×10^3^)	optimum *k*	*a*	*b*	*r*^2^	*Mammuthus* vol (m^3^)	*Mammuthus* mass (kg)	*Megatherium* vol (m^3^)	*Megatherium* mass (kg)
1	0.449	7.095	1.003	0.976	3.092	3758.5	3.097	3764.6
5	0.427	7.012	1.001	0.976	3.379	3803.1	3.400	3826.5
10	0.427	6.993	1.002	0.976	3.499	3829.0	3.476	3804.3
15	0.427	6.976	0.997	0.976	3.528	3782.6	3.514	3768.0
30	0.427	6.957	0.993	0.975	3.561	3729.0	3.577	3745.0
60	0.427	6.945	0.992	0.975	3.595	3709.8	3.613	3727.9
125	0.427	6.932	0.989	0.975	3.609	3664.3	3.652	3706.7
250	0.427	6.926	0.989	0.975	3.618	3651.0	3.662	3695.4
500	0.427	6.922	0.988	0.975	3.629^a^	3635.4^a^	3.700	3705.7

^a^Prior to downsampling, the original dataset for *Ma. primigenius* comprised only 490×10^3^ points.

In terms of choice of reference length, relative to alternative metrics, our measure of *l*_ref_ (mean distance of points from centroid) was closely related to overall body mass (*r*^2^=0.91). This compared favourably to other metrics such as maximum skeleton height (*r*^2^=0.61), bounding box diagonal length (*r*^2^=0.79) and maximum length (*r*^2^=0.87). We also found shoulder height and hip height to be relatively poorly correlated to mass in this sample (*r*^2^=0.72 and 0.64, respectively). We therefore consider our choice of reference length to be justified in this instance, and to be the preferred means of size-normalizing values of *α* across a comparative dataset.

**Figure 5.**
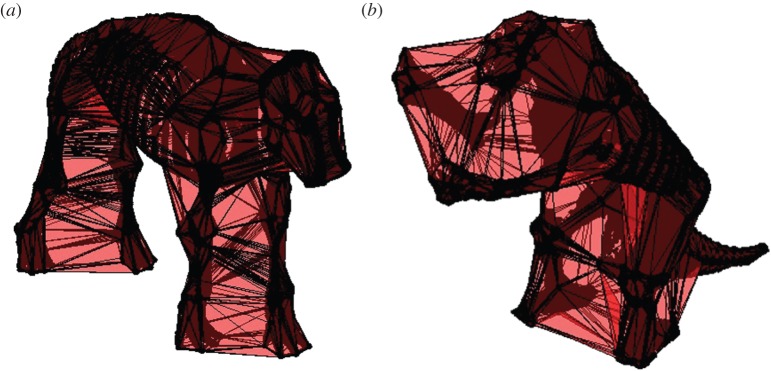
(*a*) Mammoth (*Ma. primigenius*) and (*b*) sloth (*Me. americanum*) *α*-shapes illustrating the fit achieved when refinement coefficient *k*=0.4274 (number of points=500 000). At this refinement coefficient, *α*-shapes join the right and left appendages within the fore- and hindlimbs, while leaving the forelimbs separate from the hind limbs. The skull of *Me. americanum* is also joined to the fore limbs.

Calculated values for *α*_vol_ and associated body mass estimates for *Ma. primigenius* and *Me. americanum* are given in table 3. Based on the densest point cloud dataset, the woolly mammoth (*Ma. primigenius*) is estimated to have weighed 3635 kg, and the ground sloth (*Me. americanum*) is reconstructed as weighing 3706 kg (figure 5).

## 4. Discussion

Here, we present a novel technique for estimating the body mass of extinct taxa using volumetric shape-fitting on near-complete skeletons. Complete fossil skeletons are rare as the fossil record is highly fragmentary, and the body mass of many iconic species must necessarily be estimated on the basis of very limited skeletal material. However, in the rare cases of exceptionally complete skeletons being preserved, volumetric mass estimation techniques offer an additional means of estimating mass which may be subsequently compared against traditional linear bivariate methods. It is essential that volumetric techniques are applied whenever completeness of the specimen allows in order to further understand the nature of any divergence between mass estimation techniques [11].

We hypothesized that *α*-shapes might be more reliably applied as a volumetric shape-fitting technique to the problem of fossil mass estimation than previously applied convex hulls [26,28,29,34]. Using a dense point cloud dataset (*n*=500 000), we identified an optimal refinement coefficient for the *α*-shape fit of *k*=0.427. When the resulting *α*-shape volumes (*α*_vol_) were regressed against body mass, we derived a predictive equation characterized by a very high correlation coefficient (*r*^2^=0.975) and low mean square error (m.s.e.=0.025) (table 2).

Compared to the results of Sellers *et al.* [26] in which the skeletons were manually subdivided and convex hulls fitted, our results are statistically equivalent (table 2). This suggests that the process of arbitrarily subdividing anatomical units such as the neck in order to achieve a tight-fitting hull *does not* introduce significant error into the relationship. Furthermore, the tighter wrapping *α*-shapes (and hence the inclusion of a much greater number of vertices into the volume calculation) do not appear to increase the predictive power of the model beyond that of the subdivided convex hull mode (table 2). Therefore, for this particular dataset, *α*-shapes perform equally well as convex hulls (on subdivided skeletons) in a mass prediction capacity. *α*-shapes also benefit from the ability to fit shapes to complete skeletons and removes the need for time-consuming manual segmentation, but does require significant parameter testing for any given dataset. Beyond *k*-values of 10, our *α*-shapes converge upon a convex hull shape fitted around the unsegmented skeleton (figure 2) and *r*^2^-values plateau between 0.91 and 0.92. Considering the extremely coarse nature of this *α*-shape fit around the body, this result confirms that much of the variation in body mass can effectively be described by the rough dimensions of the bounding box fitted to the extremities of the articulated skeleton.

While the *α*-shapes methodology removes the need for manual segmentation, the computer processing time required for the shape-fitting stage is considerably greater than that of convex hulling. On a standard dual-core workstation with 8GB RAM, the calculation of 200 *α*-shapes of increasing refinement coefficient for a single skeleton comprising 500 000 points took on average 68 min. Considering we repeated this process 10 times per skeleton, and included a dataset of 14 species, this results in considerable processing time. By contrast, when the dataset was downsampled to 10 000 points per skeleton, processing time was reduced to less than 1 min per skeleton. This is still markedly slower than convex hulling however, which requires less than 1 s per skeleton.

The dataset considered here comprises museum-mounted skeletons, all articulated in a quadrupedal pose. The posture in which a skeleton is mounted is a decision taken by museum preparators and researchers, and should be grounded in knowledge of the animal's anatomy and constrained by the articulatory surfaces of the skeleton. That being said, some degree of uncertainty is undoubtedly incorporated into skeletal re-articulations owing to the absence of soft tissues [35] and/or damage to the specimen. When performing computational shape-wrapping on whole skeletons (as conducted here), we might therefore expect our results to be negatively affected by uncertainty in the posing of museum mounts. Consequently, it follows that *α*-shapes calculated using much finer refinement coefficients (*k*) in which limb elements are wrapped separately (figure 2*b*) might be predicted to perform better than those in which multiple limb elements are meshed together using very coarse values for *k* (figure 2*c*).

Yet, here we find that maximum *r*^2^ and optimum *k* describes a shape in which the appendages within the fore- and hindlimbs are joined together, while the forelimbs themselves remain separate from the hindlimbs (figure 2*c*). The volume occupied by the space between appendages is therefore incorporated into the mass prediction equation. Interestingly, this model performs better (in terms of *r*^2^ and m.s.e.) than that in which all limbs are isolated. This might suggest that the spacing of the left and right limbs from one another *contains a body mass signal.* This result could be attributed to the phenomenon of postural scaling in which larger mammals have been found to adopt a more upright posture at midstance in order to mitigate size-related increases in peak bone stress during locomotion [36].

**Figure 6.**
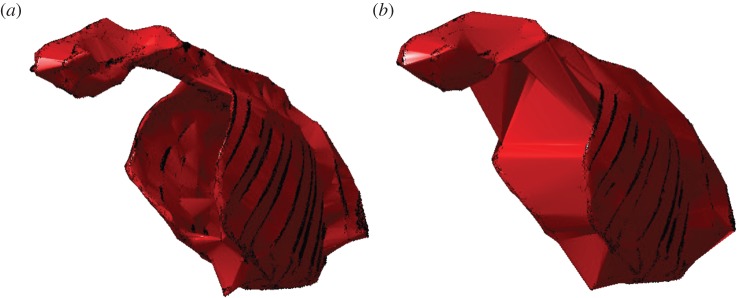
Camel ribcages at two refinement coefficients: (*a*) *k*=0.157 and (*b*) *k*= 0.427. These correspond to the two peaks in *r*^2^ for the regression fits (figure 2*b*,*c*). At lower values of *k*, the *α*-shape passes internal to the ribcage and has an overall tight fit. At the higher coefficient (which yields the best-performing predictive equation), the *α*-shape does not pass within the ribcage and has a coarser fit to the point cloud.

We consider this unlikely however, as evidence of postural scaling in modern mammals comes predominantly from the sagittal plane (‘crouched’ to erect) as opposed to the transverse plane (‘sprawling’ to erect) (although see [34]). Additionally, the dataset from Sellers *et al.* [26] is heavily skewed towards ungulates and only includes individuals greater than 80 kg in body mass, and it is questionable whether any postural signal would be discernable across these individuals. Rather, we suggest this effect (the joining together of appendages producing the best-performing predictive equations) may instead be an artefact of an optimal *k*-value sufficiently large so as to envelop the entire ribcage, as opposed to the situation in figure 2*b* in which the pivoting ‘sphere’ passes internally to the ribcage (figure 6). Additionally, we find a distinct decrease in *r*^2^ (minimum value=0.95) between these two peaks in *r*^2^ (figure 2*b*,*c*) associated with a *k*-value of 0.223 (figure 2). This corresponds to a region of *k*-values where some individuals from the modern dataset are fitted with *α*-shapes that partially join together the appendages within the fore- and hindlimbs, as well as partially passing inside the ribcage. The varying degree to which the legs are joined and ribcage enveloped are assumed to contribute to the drop in *r*^2^.

While the predictive model based upon the coarsest point cloud density (*n*=1000) was characterized by the highest value of *r*^2^, the difference in model performance between coarsest and finest datasets was extremely small (*r*^2^=0.976 and *r*^2^=0.975, respectively). We therefore regard the *α*-shape methodology presented here as being insensitive to the point cloud density of the calibration dataset (provided each individual within the modern dataset is sampled at an equal density), and consider it prudent to conserve as much of the original model data as possible. In the following discussion, we therefore consider only the results derived from the densest point cloud dataset (*n*=500 000) when moving on to use the predictive model.

When applied to fossil species, the *α*-shape equation derived here predicts body masses for the woolly mammoth (*Ma. primigenius*) and giant sloth (*Me. americanum*) of 3635 and 3706 kg, respectively. Compared to the larger steppe mammoth (*Mammuthus trogontheri*), southern mammoth (*Mammuthus meridionalis*) and Colombian mammoth (*Mammuthus columbi*), the woolly mammoth appears to have overlapped considerably in body size with extant proboscideans. A volumetric reconstruction of a very large specimen on *Ma. trogontheri* has yielded a mass estimate of 10 400 kg [37], while Christiansen [38] estimated the body mass of *Ma. meridionalis* and *Ma. columbi* (then *Mammuthus imperator*) as 6724–10 369 kg and 5215–9093 kg, respectively. By contrast, on the basis of long bone dimensions (length, diameter and circumference) of extant proboscideans, Christiansen [38] reconstructs several specimens of *Ma. primigenius* as ranging between 3058 and 6170 kg, inclusive of the 3635 kg estimated here.

Previous efforts at reconstructing the mass of extinct proboscideans have also used the relationship between body mass and shoulder height in extant taxa [39]. However, variation in overall bodily shape between modern *Elephas* and *Loxodonta* result in marked differences in body mass for any given shoulder height [38]. Furthermore, concerns have been raised regarding the uncertain positioning of the scapula on mounted skeletons of *Mammuthus* [37]. Both convex hulling and the *α*-shapes technique presented here circumvent any potential issues associated with modern taxa being particularly slim or heavily built for a given shoulder height. However, our results are sensitive to the mounting of the skeleton. The positioning of the scapula is especially difficult to constrain owing to a lack of osteological correlates. In articulated mammal skeletons, the scapula is often attached directly to the ribcage for ease of mounting. In reality, shoulder muscles such as the serratus anterior and subscapularis run between scapula and the ribcage, and the scapula sits some distance from the ribs. While convex hull shapes are defined by the positioning of a small number of extremities (such as the inferior angle of the scapula), the *α*-shapes technique presented here allows for a suite of shapes to be fitted to the skeleton, ranging in refinement from fine to coarse. Shapes defined by lower values of *k* are characterized by a tighter fit to the skeleton and are expected to be less sensitive to uncertainty in the positioning of skeletal extremities such as the scapula. Yet, future work should aim to further constrain scapula placement in order to improve museum mounts, digital reconstructions and subsequent biomechanical analyses of the forelimb.

Broadly speaking, we may be confident in the articulation of *Me. americanum* and *Ma. primigenius* mounts due to the wealth of available fossil material, high preservation quality and occurrence of modern closely related species. However when dealing with highly fragmentary material, fossils known to have undergone taphonomic deformation, or fossil species for which no close modern relative exists, it will be necessary to conduct additional sensitivity analyses to quantify the effect of articulation on resulting mass estimates. This is particularly the case when undertaking volumetric reconstructions of dinosaur species, and previous work has benefitted from the use of sensitivity analyses to rigorously bracket mass estimates with upper and lower bound volume estimates [11,24,40].

In addition to uncertainties regarding individual positioning of skeletal elements, the overall posture of the animal may influence calculated body mass. In our dataset, all specimens were standing on four limbs, with the exception of the bipedal pose of *Me. americanum* (figure 5*b*). For a given value of *k*, this change in posture resulted in some differences in *α*-shape fit for the sloth relative to other skeletons. In particular, at optimal *k*, the skull of the sloth was conjoined to the forelimbs (figure 5*b*), a feature not seen in the other specimens. The aim of the *α*-shapes method is to minimize any subjectivity introduced by re-posing and segmenting skeletons, and we consider these advantages to outweigh the potential for some increased sensitivity to posture. However, it is important to emphasize that this technique may have some sensitivity to skeletal mount, and we recommend future users visually inspect the resulting fitted *α*-shapes in order to understand how posture interacts with refinement coefficient to produce a given fit.

To estimate the mass of *Me. americanum*, Fariña *et al.* [41] applied a set of 44 allometric equations based on a modern database of craniodental and appendicular dimensions, and obtained estimates spanning 524–97 417 kg. In particular, calibration equations based upon hindlimb transverse dimensions produced extremely high mass estimates, again highlighting the susceptibility of mass predictions based on single linear dimensions to biasing by unusually robust or gracile elements. When considered in combination, estimations ranged from 6265 kg (arithmetic mean), 2543 kg (geometric mean), 2903 kg (median), 2896 kg (mode). Using a volumetric sculpting technique, Casinos [42] calculated a body mass of 3800 kg for the ground sloth, remarkably similar to the 3706 kg presented here. The close agreement between previous sculpture-based mass predictions and the *α*-shape value calculated here therefore suggest our volumetric technique is providing intuitively reasonable estimates for body mass of *Me. americanum*.

Here, we present both a new volumetric method, new predictive equations and MATLAB code, for reconstructing the body mass of mammals on the basis of their articulated skeletons. Our calibration equations are characterized by high correlation coefficients and low m.s.e., and have subsequently been applied to problem of mass estimation in extinct members of the Quaternary mammalian megafauna. The *α*-shapes technique benefits from the ability to define a suite of fitted shapes and predictive models for a given dataset, from which the best-performing may be selected and subsequently applied in a mass estimation capacity. The equation presented here is based upon a modern mammalian dataset weighing in excess of 80 kg and is heavily biased towards ungulates and, to a lesser extent, proboscideans. Our application of the *α*-shapes predictive model to *Ma. primigenius* is therefore entirely justifiable, and the close agreement found between previous volumetric mass estimates of *Me. americanum* and those presented here suggest the model may also be reliably applied to xenarthrans. However, in order for the *α*-shapes technique to be applied to other fossil groups of interest (such as theropod dinosaurs, marine reptiles or early hominids), alternative modern calibration datasets would be required. Furthermore, the ‘optimum’ refinement coefficient (*k*) producing the best-performing predictive equations is specific to the calibration dataset and will probably vary as a function of the species included, and potentially the imaging techniques employed to generate three-dimensional models. As such, optimum *k* should be recomputed when undertaking future analyses on new datasets.

When fitting *α*-shapes to the modern OUMNH dataset, antlers were missing from some museum skeletons, and we therefore manually removed antlers and tusks from the models prior to further analysis for consistency. The *α*-shapes technique presented here does however accommodate for the inclusion of unusual accessory structures such as horns and antlers, and their removal is not a prerequisite. *α*-shapes could be used to derive a cervid-based predictive model to be applied to fossil deer, for example, without the removal of antlers. However, a more cautious approach is needed if the predictive model is to be applied to a distantly related fossil group possessing unusual structures, such as the armour of thyreophoran dinosaurs. In instances when fossil accessory structures are not homologous to those present in the modern dataset, we suggest a more judicious approach followed by Brassey *et al.* [11] in which the features are removed and surfaced separately, and resulting masses added to those predicted via shape-fitting.

For the woolly mammoth and giant sloth included here, we provide a single straightforward estimate of body mass. For the purpose of investigating temporal or spatial patterns in body mass, for example [10,43], such values are entirely adequate. Likewise, if force (as a multiple of body mass) is required for finite-element analysis [28], a single value of mass may be sufficient. However, for some biomechanical studies, a simple scalar value for body mass is only part of the solution. For the purpose of multi-body dynamic analyses (MBDA), the distribution of said mass around the skeleton is also required, as quantified by the COM and body segment inertial properties. In the case of digital sculpting techniques such as NURBs curves, which actively seek to reconstruct the fleshed-out surface contours of the individual, such properties can be estimated. However, when applying less subjective shape-fitting techniques such as convex hulling and *α*-shapes, it is not yet clear how the distribution of volume in the fitted shape corresponds to the distribution of volume within the live animal. Sellers *et al.* [29] fit convex hulls around the skeleton of *Argentinosaurus* prior to MBDA and expand the hulls in order to achieve a 20% increase in volume, and hence mass. The original application of convex hulling found that approximately 20% of the mass of the animal was lost through the process of fitting minimum-wrap shapes [26], and must therefore be recovered by ‘re-inflating’ the hulls. However, the decision as to where the mass should be added is somewhat arbitrary, and the same would undoubtedly be true if we were to attempt to infer segment properties from fitted *α*-shapes. Future work should aim to quantify the relationship between fitted geometric shapes and overlying soft-tissue contours, as determined from computed tomography imaging of extant taxa. Until such data are available, the application of *α*-shapes in its current form should be restricted to straightforward mass estimation.

Here, we have contributed to the burgeoning body of research aiming to improve body mass estimates using three-dimensional volumetric models. An ongoing concern in the field of palaeontology more generally is the use of the present to reconstruct the past. In doing so, there is the potential to ‘condemn[s] the past to be like the present’ [44, p. 532], and for the animals we reconstruct to be averaged ‘everyanimals’. With regards to body mass estimation, applying a relationship between isolated skeletal elements and mass found in modern animals to reconstruct long extinct species (often with widely differing body plans) can seem particularly questionable. We consider *α*-shapes to perhaps be less vulnerable to this ‘everyanimal’ phenomenon, as the underlying predictive relationship between skeletal volumes and body mass may reflect more fundamental biomechanical or physiological processes limiting the quantity of soft tissue that can feasibly be accommodated around a given skeleton. Indeed, the close agreement between our *α*-shapes mass estimate of *Me. americanum* and those previously calculated from scale models suggests this technique is capable of providing reasonable estimates for otherwise ‘peculiar’ forms [45].

That being said, the application of this technique to fossil taxa does still assume an average ‘modern’ volume of soft tissue to be held beyond the bounds of the reconstructed *α*-shape, and does not account for any potential variability. Unfortunately, in this respect, we are little more informed with regards to modern species than we are with extinct taxa. There is a notable paucity of data describing interspecific variability in body composition among modern taxa, and even less regarding variation in body mass within species and within the individual over time. Volumetric approaches such as the one detailed here are providing us with increasingly sophisticated techniques for modelling the three-dimensional geometry of extinct species. A similar effort is now required to shift the focus back onto the present and improve the quality of modern data upon which we based our reconstructions.
